# Interpretable Machine Learning with SHAP Identifies Key Biomarkers in a Multi-Factorial Spectrum of Age-Related Neurological and Metabolic Conditions

**DOI:** 10.3390/ijms27041805

**Published:** 2026-02-13

**Authors:** Daniil V. Artamonov, Polina I. Popova, Ekaterina A. Korf, Natalia G. Voitenko, Alisa A. Chernysheva, Pavel V. Avdonin, Richard O. Jenkins, Nikolay V. Goncharov

**Affiliations:** 1 Group of Theoretical Chemistry, N.D. Zelinsky Institute of Organic Chemistry of Russian Academy of Sciences, Leninsky Prospect 47, Moscow 119991, Russia; 2 Infochemistry Scientific Center, National Research University ITMO, Kronverksky Ave. 49, Building A, St. Petersburg 197101, Russia; 3 St. Petersburg State Budgetary Healthcare Institution “City Polyclinic No. 107”, St. Petersburg 195030, Russia; 4 Sechenov Institute of Evolutionary Physiology and Biochemistry, Russian Academy of Sciences, pr. Torez 44, St. Petersburg 194223, Russia; 5 Department of Computational Biology, Sirius University of Science and Technology, Olympic Ave. 1, Sirius 354340, Russia; 6 Koltsov Institute of Developmental Biology, Russian Academy of Sciences, 26 Vavilov St., Moscow 119334, Russia; 7 Leicester School of Allied Health Sciences, De Montfort University, The Gateway, Leicester LE1 9BH, UK; 8 Research Institute of Hygiene, Occupational Pathology and Human Ecology of the Federal Medical Biological Agency, p.o. Kuz’molovsky bld.93, St. Petersburg 188663, Russia; 9 Department of Biological Chemistry, Petersburg State Pediatric Medical University, Ministry of Health of the Russian Federation, Litovskaya St. 2, St. Petersburg 194100, Russia

**Keywords:** SHAP (Shapley Additive exPlanations), machine learning, biomarker discovery, heteroscedasticity, Welch’s ANOVA, age-related diseases, vascular dementia, type 2 diabetes, classification, feature selection

## Abstract

Vascular and metabolic disorders in the elderly—including acute ischemic stroke (AIS), chronic cerebral circulation insufficiency (CCCI), type 2 diabetes mellitus (DM), and subcortical ischemic vascular dementia (SIVD)—pose a major diagnostic challenge due to their reliance on multi-parameter blood chemistry. In this study, 49 biochemical features were analyzed within a cohort of 120 patients. The application of variance-aware statistical testing revealed that several features (e.g., Fe, Transf, RDW%, LDL) exhibited statistically significant heterogeneity of variance (*p* < 0.05), which is known to distort standard ANOVA inference. While standard machine-learning (ML) classifiers demonstrated variable performance across clinical groups, a gradient boosting model with restricted tree depth (max depth = 3) achieved high discriminative accuracy, yielding F1-scores between 0.87 and 0.96 across all five clinical classes. Through the use of Shapley Additive Explanations (SHAP), key stable biomarkers including iron (Fe), transferrin, and glucose were identified as having synergistic interactions in model predictions. A comparative analysis of feature importance ranks indicated consistency between statistical significance and SHAP values, with Spearman correlation coefficients reaching 0.53 for groups 1–2 and 0.59 for groups 1–5. Conversely, unsupervised KMeans clustering (k = 5) revealed a poor correspondence with clinical labels, yielding an Adjusted Rand Index (ARI) of 0.198 and Normalized Mutual Information (NMI) of 0.286. These results underscore that statistical structures in biochemical data do not always map to meaningful clinical categories and advocate for the adoption of variance-aware workflows and interpretable ML to enhance diagnostic reliability in aging populations.

## 1. Introduction

Diagnostic error is commonly defined as a missed, delayed, or incorrect diagnosis and is considered one of the most serious threats to patient safety [1]. A large-scale study of patients transferred to intensive care units or who died in hospitals found that diagnostic errors were present in nearly one-quarter of cases. Approximately every fifteenth death was associated with a diagnostic error. These errors were most often linked to delays in diagnosis and incorrect selection of diagnostic tests, leading to worse outcomes and high mortality rates [2]. In 2010–2011, an estimated 40,000 adult patients died annually in the United States due to misdiagnosis in intensive care units and emergency departments, while 10 years later, this number had risen to 250,000 [3,4]. The leading causes of death are vascular disease and infections. The implementation of artificial intelligence (AI) systems in clinical diagnosis represents a promising direction for developing reliable support tools that can improve the accuracy of diagnostic decisions, optimize examination time, and reduce the risk of medical errors [5].

However, beyond the emergency departments, vascular and metabolic disorders represent a substantial clinical and public health burden in the aging population, with acute ischemic stroke (AIS), chronic cerebral circulation insufficiency (CCCI), prediabetes, type 2 diabetes (DM), and subcortical ischemic vascular dementia (SIVD) being among the most prevalent and debilitating conditions affecting elderly individuals [6]. AIS, a major cause of mortality and morbidity in the elderly, has become increasingly prevalent in recent years, with ischemic stroke accounting for approximately 70–80% of all stroke cases and demonstrating age-dependent increases in incidence, particularly among individuals aged 85 years and older [7]. Chronic cerebral vascular insufficiency and cerebral small vessel disease contribute substantially to cognitive decline and neurological dysfunction through progressive deterioration of small penetrating arteries, leading to white matter hyperintensities, lacunar infarctions, and impaired cerebral autoregulation [8]. Prediabetes and DM affect a large proportion of elderly individuals, with up to 70% of patients with prediabetes eventually progressing to diabetes [9]. In elderly patients with DM, macrovascular complications including coronary heart disease (42.4%), stroke (11.4%), and chronic obliterating disease of the lower limb arteries (10.1%) are highly prevalent, alongside microvascular complications such as retinopathy (64.9%) and polyneuropathy (90.7%) [10]. Subcortical ischemic vascular dementia, often referred to as Binswanger’s disease in its severe form, represents the most common subtype of vascular dementia and is characterized by confluent white matter hyperintensities, multiple lacunar infarcts, and progressive cognitive decline with prominent executive dysfunction, psychomotor slowing, and gait disturbances [11]. The clinical significance of these interconnected vascular and metabolic disorders extends beyond individual morbidity and mortality, as cerebral small vessel disease has been identified as a critical contributor to approximately 50% of dementia cases globally and serves as both an initiator and amplifier of neurodegenerative pathology [12,13]. Biomarkers serve as a fundamental basis for optimizing primary screening by providing enhanced criteria for early detection and risk stratification. They contribute to the development of personalized approaches to diagnosis and prevention in oncology, neurodegenerative, and metabolic disorders [14].

Classical analysis of variance (ANOVA) remains a widely used statistical method for comparing mean outcomes across multiple treatment or diagnostic groups in biomedical research. However, a fundamental assumption of ANOVA—homogeneity of variance (homoscedasticity)—is frequently violated in clinical and biochemical datasets, leading to potentially spurious results and compromised statistical inference. When the assumption of equal variances is violated, the standard ANOVA F-test becomes unreliable, with Type I error rates deviating substantially from the nominal significance level [15]. Heteroscedasticity in biomedical data is particularly problematic because it distorts estimated standard errors, rendering confidence intervals inaccurate and hypothesis tests unreliable, which can lead to incorrect conclusions about treatment efficacy or diagnostic group differences [16]. The presence of heteroscedasticity is common in clinical trials and multi-site studies, where inherent biological variability, differences in measurement precision across subgroups, and varying sample sizes combine to produce unequal variances that systematically reduce statistical power to detect true treatment effects [17].

Importantly, Welch’s ANOVA maintains comparable or even superior statistical power to classical ANOVA when variances are equal, meaning that it can be used routinely without concern for power loss, effectively eliminating the need to test for homogeneity of variance as a prerequisite for analysis [18]. In clinical trials and biomedical research settings, Welch’s ANOVA has been successfully applied to analyze biochemical markers and clinical endpoints where unequal variances are anticipated; for instance, in studies examining biomarker changes in knee synovial fluid from patients with anterior cruciate ligament injuries, Welch’s ANOVA enabled valid comparison of cytokine concentrations across injury groups despite significant heteroscedasticity [19]. Comparative studies have demonstrated that Welch’s ANOVA consistently provides more accurate Type I error control and better power performance than both the traditional ANOVA F-test and several other alternatives under conditions of heteroscedasticity, establishing it as a preferred method for analyzing clinical and experimental data with unequal variances [20].

Machine learning (ML) algorithms have demonstrated substantial efficacy in classification and diagnosis tasks based on biochemical and clinical data, with various supervised learning methods including linear discriminant analysis (LDA), random forest (RF), support vector machines (SVM), logistic regression (LR), k-nearest neighbors (kNN), and decision trees (DT) showing strong performance across diverse clinical applications [21]. Random forest, an ensemble learning method that constructs multiple decision trees and aggregates their predictions, has emerged as one of the most widely applied ML techniques in medical research with particular success in handling high-dimensional datasets, such as electronic health records, and demonstrating robustness to missing data and resistance to overfitting [22]. Investigations into oncological applications have shown that SVM and DT approaches can yield accuracies exceeding 97% when differentiating between benign and malignant lesions, as seen in skin cancer detection studies, underscoring the capacity of these algorithms to handle high-dimensional data while maintaining exceptional sensitivity and specificity [23]. Logistic Regression continues to play a vital role in clinical prediction models by providing a robust probabilistic framework that can integrate multiple covariates to predict disease risk, offering interpretable coefficients that directly translate into clinical risk estimates [24]. Support vector machines, employed in 32% of contemporary studies, excel in handling high-dimensional data and nonlinear relationships between features, demonstrating particular utility in genomic data analysis, medical imaging classification, and precision medicine applications where complex patterns must be identified from multi-modal datasets [25]. K-nearest neighbors algorithms have been successfully applied to medical diagnosis tasks, particularly in scenarios where local patterns in feature space are informative for classification, although their performance can be sensitive to the choice of distance metric and the number of neighbors [26]. Furthermore, the flexibility of these ML algorithms to adapt to high-dimensional and heterogeneous data environments underscores their pivotal role in modern clinical diagnostics, where they not only enhance predictive performance but also contribute to a deeper understanding of underlying disease mechanisms [27].

Shapley Additive Explanations (SHAP), a framework grounded in cooperative game theory for explaining machine learning predictions, has become a mainstream approach in data science for interpreting complex models and enhancing the clinical utility of ML-based diagnostic and prognostic systems. SHAP values assign each feature an importance value for a particular prediction by fairly distributing the ‘payout’ among the features, thus providing explainability locally for individual predictions as well as globally across datasets [28]. Recent studies integrating convolutional neural networks with attention mechanisms and SHAP for personalized health monitoring have achieved remarkable accuracies of 97.86%, with SHAP providing both population-level feature importance rankings and patient-specific explanations through force plots and dependence visualizations, which enable clinicians to understand why specific predictions were made for individual patients [29]. The XGBoost model combined with SHAP analysis accurately predicted lymph node metastasis in gastric cancer patients. SHAP values identified inflammatory indices and peripheral lymphocyte subpopulations as top contributors, with SHAP values ranging from 0.15 to 0.32 on average. This approach enhanced model interpretability and achieved high predictive accuracy (AUC > 0.85) [30]. In acute heart failure prediction, XGBoost models combined with SHAP analysis have demonstrated superior performance with AUC values of 0.817 for in-hospital mortality and 0.766 for worsening heart failure, while SHAP clustering has enabled risk stratification by forming interpretable patient clusters with similar mortality and complication rates [31].

This study focuses on comparing one-way ANOVA and Welch’s ANOVA for selecting biochemical features across patient groups, followed by the evaluation of ML classifiers (LDA, LR, kNN, DT, RF, SVM) and interpretation using SHAP. The objective is to develop ML models for a personalized diagnosis of cardiovascular diseases, providing enhanced accuracy and interpretability of results.

## 2. Results

### 2.1. Data Analysis

Statistical analysis of the dataset provides an essential foundation for understanding the distribution and variability of biochemical features across patient groups. Initial screening was performed with classical statistical methods to identify features that differ between groups and to inform subsequent feature selection for machine learning. In particular, assessment of variance homogeneity and between-group differences is important because these characteristics influence both the interpretability and predictive performance of supervised models.

**Figure 1:**
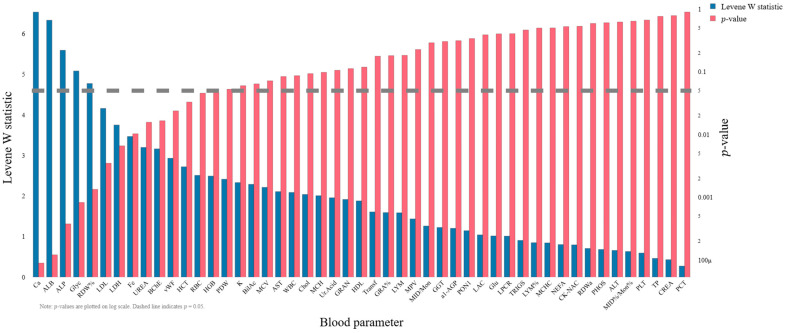
Levene test results for biochemical parameters across patient groups. The blue bars represent the Levene W statistic, while the red bars indicate *p*-values (logarithmic scale). The heavy dashed horizontal line denotes the *p* = 0.05 significance threshold. A *p*-value below this line (*p* < 0.05) indicates that the assumption of homogeneity of variances is statistically violated (heteroscedasticity). In this dataset, parameters to the left of PDW (e.g., Ca, ALB, ALP, Glyc, RDW%, LDL, LDH, Fe, UREA, BChE, vWF, HCT, RBC and HGB) demonstrate significant variance heterogeneity, necessitating the use of Welch’s ANOVA to ensure robust statistical inference.

To test variance homogeneity, Levene’s test was applied to each biochemical parameter. Figure 1 shows the Levene W statistics and corresponding *p*-values (right axis, log scale) for all parameters. Several features exhibited statistically significant heterogeneity of variance (*p* < 0.05, e.g., Fe, Transf, RDW%, LDL, LDH), indicating violation of the equal-variance assumption required by classical one-way ANOVA.

**Figure 2:**
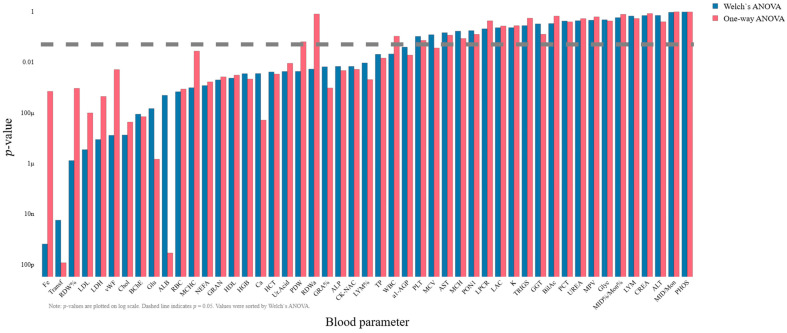
Comparison of *p*-values obtained from classical one-way ANOVA and Welch ANOVA for all blood parameters. The bar chart displays Welch ANOVA results (blue bars, left group) and one-way ANOVA results (red bars, right group) for each parameter. *p*-values are plotted on a logarithmic scale to enhance visibility of small values. The dashed horizontal line at *p* = 0.05 indicates the threshold for statistical significance. Features are sorted according to Welch ANOVA *p*-values to facilitate comparison. This visualization highlights discrepancies between the two methods, illustrating the impact of variance heterogeneity on the assessment of statistical significance.

Due to the observed heteroscedasticity, the results of classical one-way ANOVA were compared with Welch’s ANOVA, which relaxes the equal-variance assumption by adjusting the F-statistic and degrees of freedom. Figure 2 presents *p*-values from both methods for all blood parameters (*p*-values plotted on a log scale and sorted by Welch’s ANOVA). The comparison highlights marked discrepancies in statistical significance for a subset of parameters.

**Figure 3:**
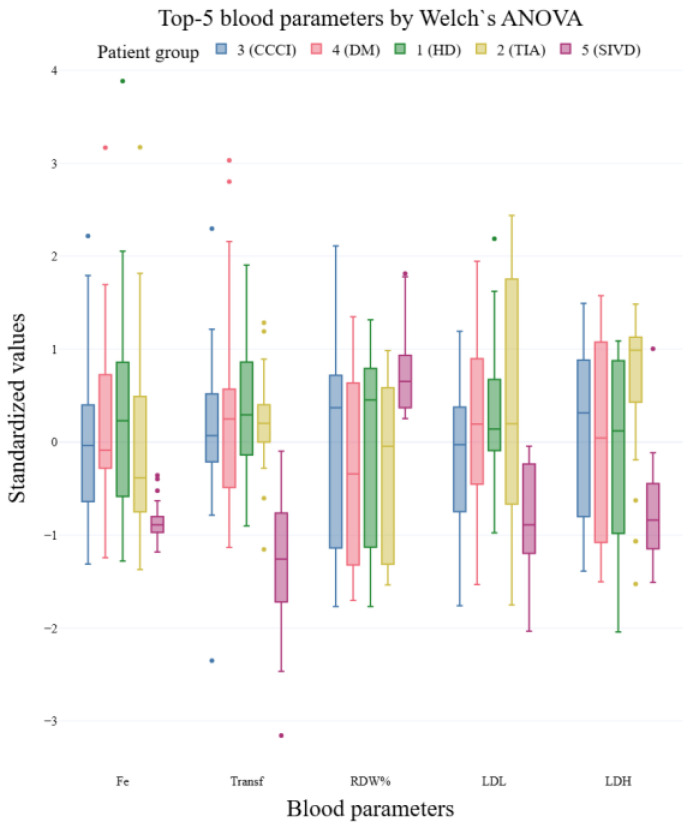
Top 5 blood parameters based on Welch’s ANOVA across patient groups.

**Figure 4:**
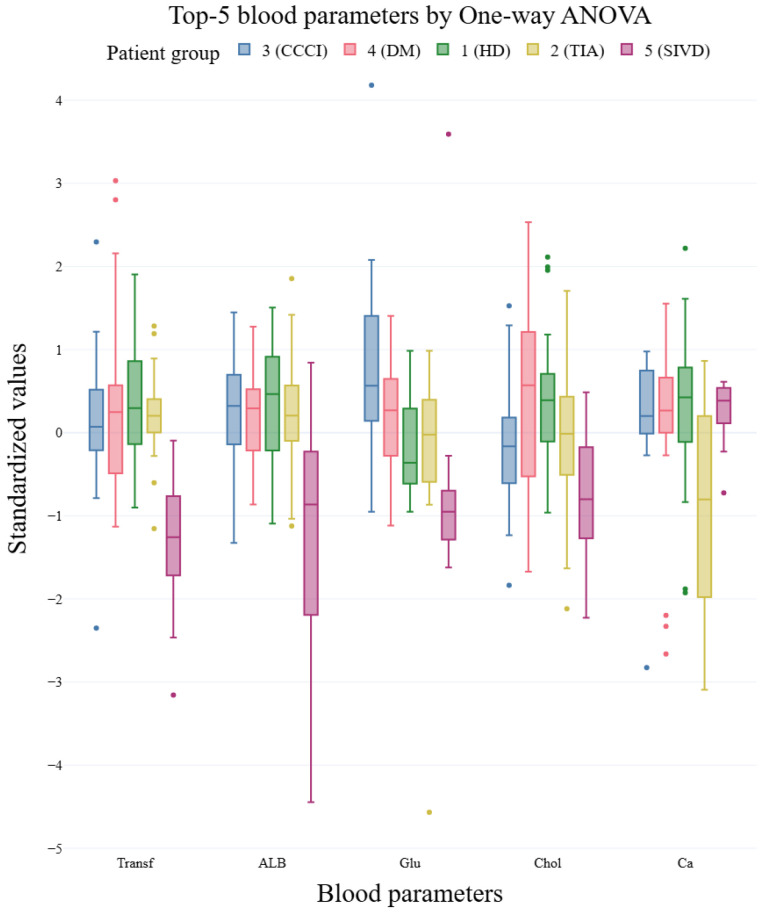
Top 5 blood parameters based on One-ANOVA across patient groups.

Notably, the divergence between methods is pronounced for several parameters (e.g., Fe, Transferrin (Transf), RDW%, Glucose, Albumin, RDWa, von Willebrand Factor (vWF), LDL). For these features, the *p*-values produced by Welch’s ANOVA differ substantially from those of classical ANOVA, reflecting the influence of unequal variances across groups. Box plots of the top 5 features ranked by Welch’s ANOVA and by one-way ANOVA are shown in Figure 3 and Figure 4, respectively. For the boxplots, feature values were standardized (z-score) prior to plotting to facilitate visual comparison of group distributions.

This phenomenon—substantial differences in ranked lists of significant features between the two ANOVA variants—indicates that heteroskedasticity materially affects significance calls. Selecting an appropriate test (e.g., Welch’s ANOVA when variances are unequal) therefore improves the reliability of feature selection, which in turn supports construction of more robust and interpretable machine-learning models.

**Figure 5:**
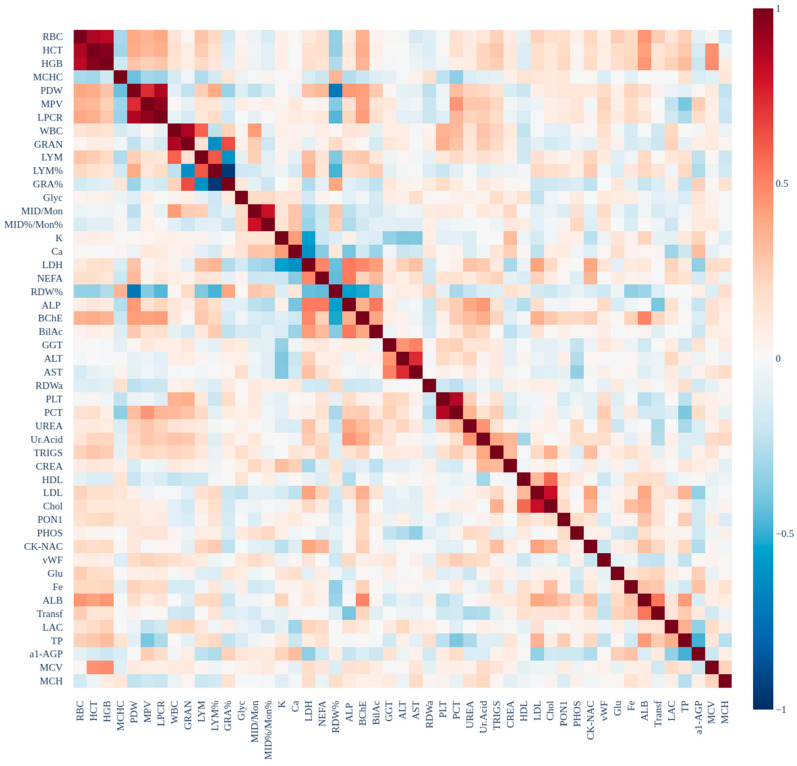
Reordered correlation heatmap of biochemical features. To enhance interpretability, variables were grouped using hierarchical clustering (Ward’s method) based on the distance metric d = 1 − ∣r∣. This approach automatically clusters biologically related markers—such as red cell indices (RBC, HGB, HCT), platelet parameters (PLT, PCT, LPCR), and lipid profiles (Chol, LDL)—revealing dense blocks of high multicollinearity (∣r∣ > 0.8). Such structural redundancy informs the subsequent use of SHAP interaction analysis to capture the synergistic roles of correlated predictors.

Multicollinearity Check: To ensure that features included in the models are not highly correlated with one another, Pearson correlation was used to examine the relationships between all features. A heatmap of the correlation matrix was generated to visualize multicollinearity, with highly correlated features (correlation coefficient > 0.8) flagged for potential exclusion from subsequent analysis to reduce collinearity and improve model performance. The correlation heatmap is shown in Figure 5.

### 2.2. Comparison of ANOVA Variants Under Heteroscedasticity

To quantitatively validate the impact of heteroscedasticity on classical ANOVA inference, five statistical tests were compared: classical ANOVA, Welch’s ANOVA, Levene’s test, Brown-Forsythe (Levene on medians), and Heteroskedasticity-Consistent Covariance Matrix Estimator (HC3) (Table S1). The comparative testing results reveal the fundamental limitations of ANOVA under violated homoscedasticity assumptions. As shown in Table S1, which presents the number of significant parameters (at α = 0.05 significance level), classical ANOVA identified 25 significant features, clearly illustrating the tendency toward Type I error inflation in the presence of unequal variances across patient groups. This inflation occurs because the standard ANOVA F-test is highly sensitive to deviations from variance homogeneity, leading to distorted statistical inference. In contrast, Welch’s ANOVA identified 26 significant parameters, demonstrating a more conservative and robust approach that maintains statistical power without compromising Type I error control even under heteroscedasticity conditions. Comparison with alternative robust methods—including Brown-Forsythe (15 significant), HC3 (27 significant), and Kruskal-Wallis (27 significant)—confirms consensus between Welch’s ANOVA and Kruskal-Wallis (Spearman ρ = 0.92), providing strong methodological justification for selecting Welch’s ANOVA as the primary feature selection method under the heteroscedastic conditions characteristic of biochemical biomarker datasets.

### 2.3. Machine Learning

A set of classical supervised classifiers was evaluated to quantify discriminative performance and the effect of different feature-selection strategies. The following models were considered: LDA, LR, kNN, DT, RF and SVM. These methods cover linear and non-linear paradigms and provide complementary perspectives on class separability and feature relevance.

Three feature sets were used in the experiments: (1) the full preprocessed feature set including all biochemical variables, (2) a subset of features selected by Welch’s ANOVA, and (3) a subset selected by classical one-way ANOVA. For both ANOVA-based selections, only features with *p*-values below 0.05 were retained. The one-way ANOVA subset included the following features: Transf, ALB, Glu, Chol, Ca, BChE, LDL, LDH, Fe, RBC, RDW%, GRA%, NEFA, LYM%, HGB, GRAN, HDL, HCT, ALP, vWF, CK-NAC, Ur.Acid, TP, a1-AGP, MCHC, and MCV. The Welch ANOVA subset consisted of: Fe, Transf, RDW%, LDL, LDH, vWF, Chol, BChE, Glu, ALB, RBC, MCHC, NEFA, GRAN, HDL, HGB, Ca, HCT, Ur.Acid, PDW, RDWa, GRA%, ALP, CK-NAC, LYM%, TP, WBC, and a1-AGP. All numerical predictors were standardized (z-score), and all preprocessing steps were performed within cross-validation folds to prevent information leakage.

Model selection and hyperparameter optimization were performed using stratified 3-fold cross-validation with an inner grid search for each classifier. Performance was summarized by per-class F1-score (reported in tables) together with macro-average F1, Precision, and Recall metrics. Detailed per-class results and aggregated metrics are provided in Table 1.

**Figure 6:**
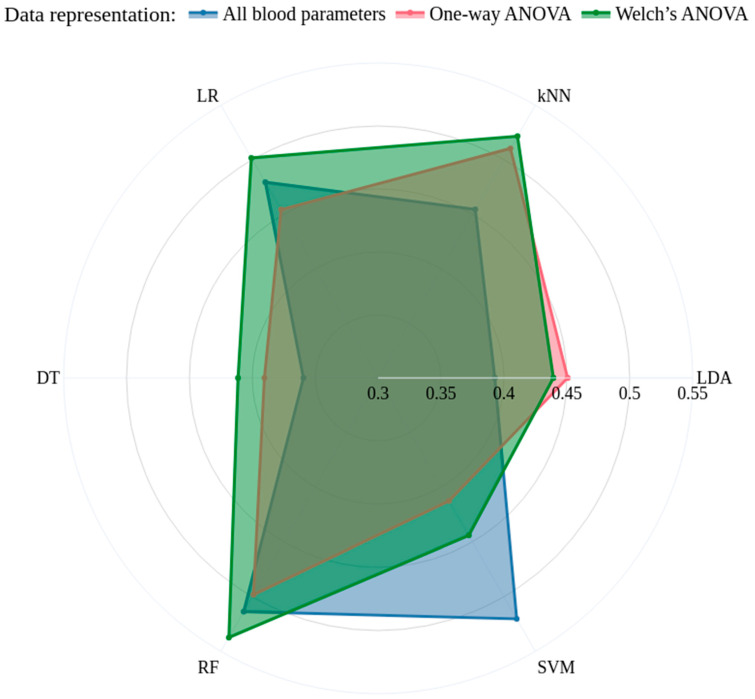
Radar (spider) plot comparing classifiers by mean per-class *F*_1_-score under three feature-selection strategies. Each axis corresponds to a classifier (LDA, kNN, LR, DT, RF, SVM); closed polygons show average *F*_1_ across classes for a given feature set: all features, features selected by one-way ANOVA, and features selected by Welch’s ANOVA. Radial scale indicates mean *F*_1_ (higher values denote better class-level performance); plot range is the [0.2, 0.55] interval used for visualization.

Figure 6 shows a radar (spider) plot of the mean per-class F_1_-score for six classifiers (LDA, kNN, LR, DT, RF, SVM) under three feature-selection strategies. Each axis represents a classifier, and the closed polygons correspond to the F_1_-score averaged across all classes for each feature set: the full set of features, features selected by one-way ANOVA, and features selected by Welch’s ANOVA. The plot provides a concise visual comparison of classifier performance across feature-selection strategies, highlighting relative differences rather than absolute F_1_ values.

### 2.4. Personalized and Interpretable Models

**Figure 7:**
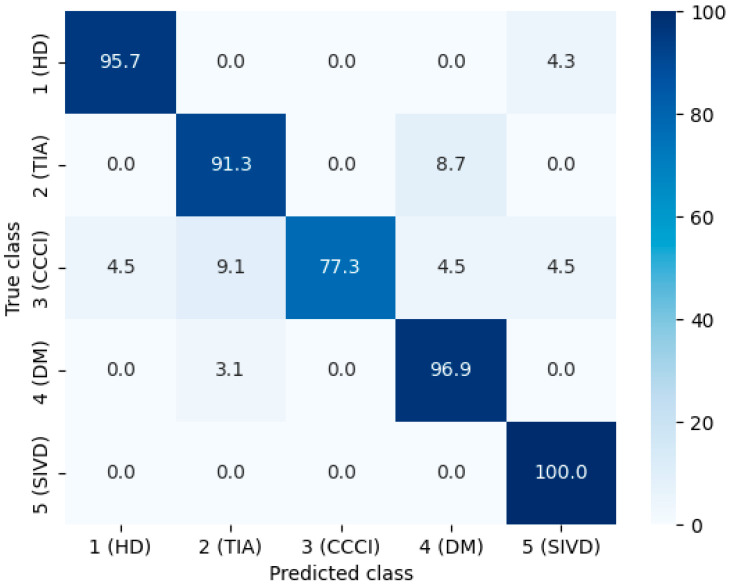
Confusion matrix of the Gradient Boosting model on the full dataset. Cell colors indicate the percentage of predictions: diagonal cells show correctly classified instances, while off-diagonal cells indicate misclassifications. Percentages are normalized per true class.

For the SHAP analysis, a Gradient Boosting model with a tree depth of 3 was used to prevent overfitting on the full dataset while maintaining interpretability. This shallow tree structure ensures that the computed SHAP values are stable and reliable. In fact, this choice is heuristic, so we obtained the learning curves for the gradient boosting classifier (Figure S1) and conducted a sensitivity analysis of the gradient boosting model (Figure S2). Tree SHAP allows for exact and computationally efficient calculation of Shapley values, capturing not only the individual contributions of each biochemical feature to the model’s predictions, but also interactions between features. As a result, the analysis provides a nuanced understanding of how combinations of variables influence patient classification and helps identify the most influential features beyond simple correlations. The model’s performance is presented in the confusion matrix (Figure 7) and summarized in Table 2.

**Figure 8:**
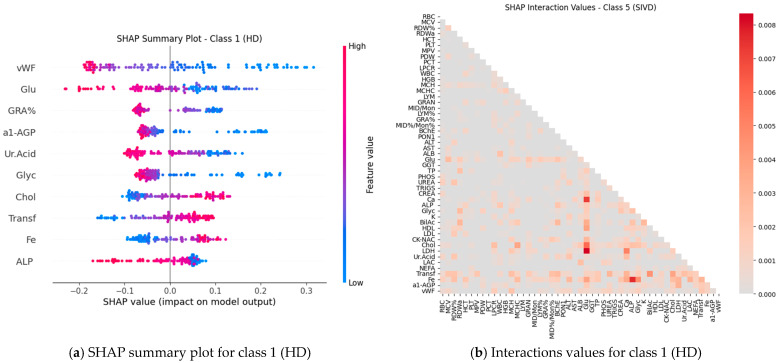
Feature contributions for class 1 (HD): (**a**) Interactions between features; (**b**) Summary of individual feature impacts.

**Figure 9:**
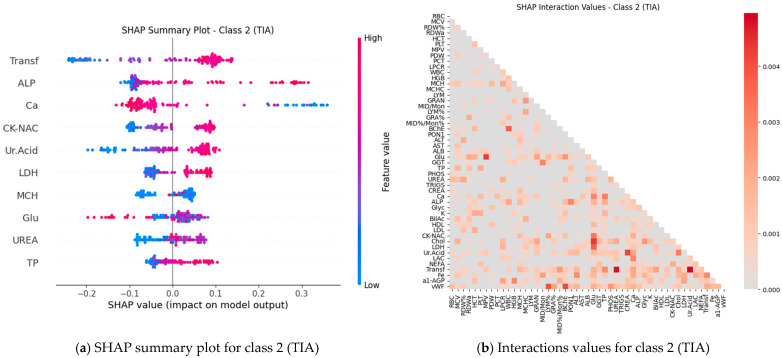
Feature contributions for class 2 (TIA): (**a**) Interactions between features; (**b**) Summary of individual feature impacts.

**Figure 10:**
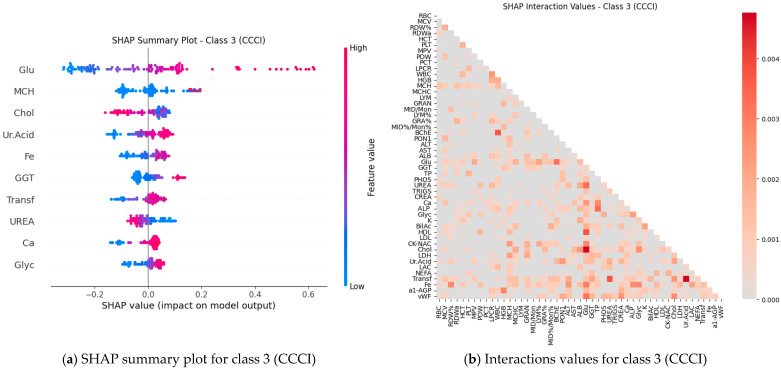
Feature contributions for class 3 (CCCI): (**a**) Interactions between features; (**b**) Summary of individual feature impacts.

**Figure 11:**
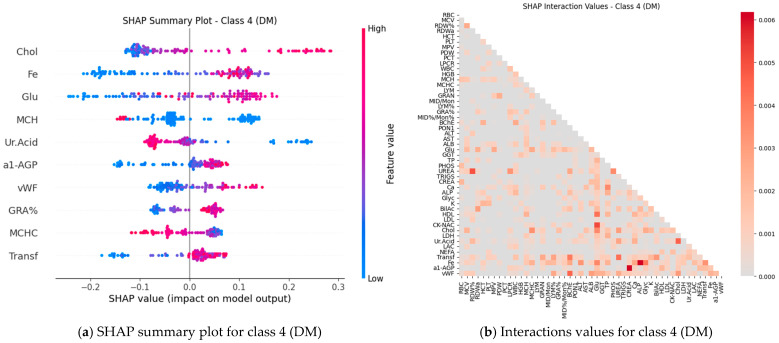
Feature contributions for class 4 (DM): (**a**) Interactions between features; (**b**) Summary of individual feature impacts.

**Figure 12:**
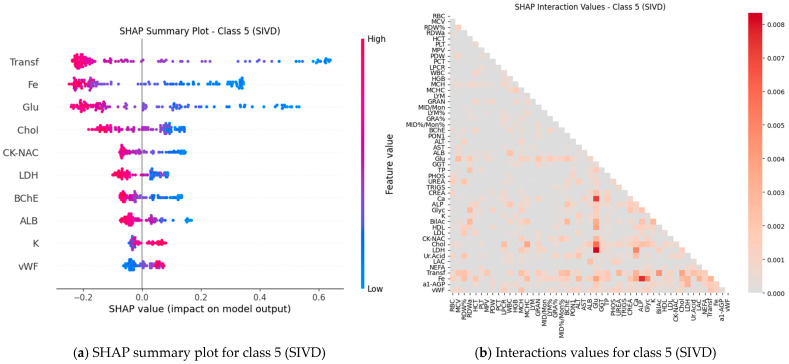
Feature contributions for class 5 (SIVD): (**a**) Interactions between features; (**b**) Summary of individual feature impacts.

Subsequently, SHAP values were computed for all features (blood test parameters) across all classes to evaluate their individual contributions to the model’s predictions. The results are presented for each class using paired plots: one showing feature interactions and the other summarizing individual feature impacts. Figure 8, Figure 9, Figure 10, Figure 11 and Figure 12 display these visualizations for classes 1 through 5, highlighting the most influential features and the strength and direction of their effects on the predicted probabilities. Features are ranked by global importance. Each dot represents a patient; red indicates a high biomarker value, while blue indicates a low value. Dots to the right of the midline increase the diagnostic probability for the respective class, whereas dots to the left decrease it.

### 2.5. Mutual Information for Feature Synergy (MI)

To further investigate feature relationships and interactions, two complementary visualizations were constructed. First, a feature interaction network was created where each feature was represented as a node. The top 50% of features were selected based on mutual information with the target variable. Edges were added between feature pairs exhibiting absolute Pearson correlations above 0.3, with edge weights proportional to correlation strength. Isolated nodes were removed, retaining only interacting features. Community detection was performed using the Louvain algorithm, and nodes were arranged in a circular layout, with cluster membership indicated by distinct pastel colors. Edge widths reflected correlation magnitude, providing an intuitive representation of clusters of features that may jointly contribute to class discrimination.

**Figure 13:**
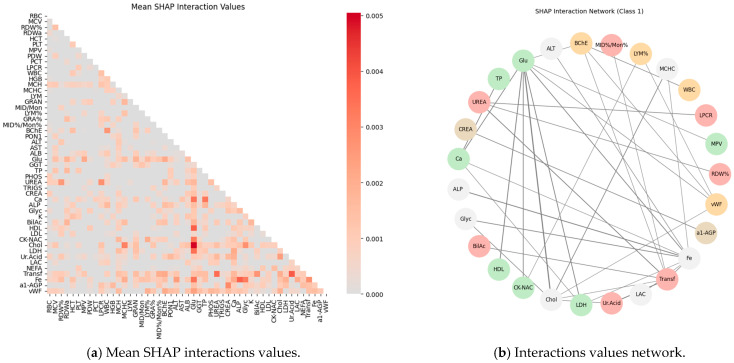
Mean SHAP Interaction Values (**a**) and Corresponding Feature Interaction Network (**b**) Across Classes.

Second, to capture interaction effects identified by the predictive model, the absolute SHAP interaction values were computed and averaged across all classes. The resulting mean interaction matrix was visualized as a heatmap (Figure 13a). This visualization highlights pairs of features whose combined effects contribute substantially to model predictions, complementing the correlation-based network by revealing non-linear and class-dependent interactions. Together, these analyses provide a multi-level perspective on both linear dependencies and model-informed synergistic relationships among features. The combined results of the feature interaction network and the mean SHAP interaction values across classes are presented in Figure 13.

### 2.6. Unsupervised Pattern Discovery

**Figure 14:**
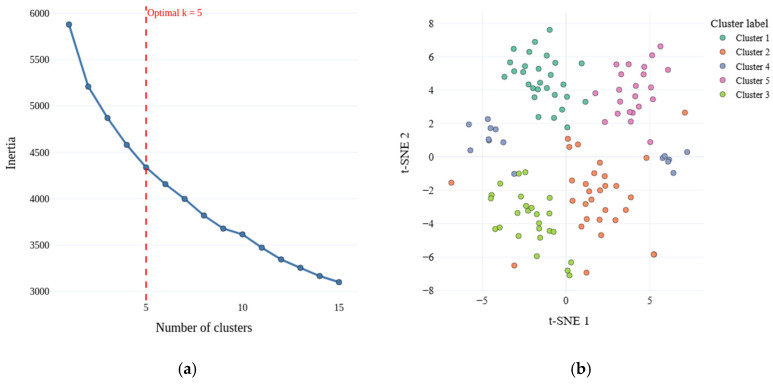
Clustering analysis of the dataset. (**a**) Elbow method used to determine the optimal number of clusters (k = 5); (**b**) Two-dimensional t-SNE projection of the data points colored by their assigned KMeans cluster.

Unsupervised clustering was performed on the full feature space using the KMeans algorithm. The optimal number of clusters was determined via the elbow method, which involves plotting the within-cluster sum of squares (WCSS) as a function of the number of clusters and identifying the point where the rate of decrease sharply diminishes. This approach suggested k = 5, coincidentally matching the number of predefined classes in the dataset (Figure 14a).

To visualize the clustering results, the high-dimensional feature space was projected into two dimensions using t-distributed Stochastic Neighbor Embedding (t-SNE), which preserves local neighborhood structures and allows for intuitive visualization of complex patterns. The resulting 2D embeddings of the clustered objects are shown in Figure 14b.

To assess the quality of the clustering and its correspondence to the ground-truth classes, the Silhouette score for the original class labels in the full feature space was first evaluated, yielding a value of −0.139. After applying KMeans clustering, the Silhouette score increased to 0.122, indicating a moderate improvement in the cohesion of the assigned groups. The agreement between the derived clusters and the true labels was further quantified using the Adjusted Rand Index (ARI = 0.198) and Normalized Mutual Information (NMI = 0.286). The ARI measures the similarity between two labels while correcting for chance, with values closer to 1 indicating strong agreement, whereas the NMI evaluates the mutual dependence between cluster assignments and true classes, also ranging from 0 (no correspondence) to 1 (perfect match). Despite the moderate improvements in these metrics, the results suggest that the clusters do not fully capture the original class structure. This discrepancy may be partly due to the high dimensionality of the feature space, which can complicate the separation of clusters and obscure intrinsic patterns in the data. The composition of each cluster with respect to the original classes is summarized in Table 3.

**Figure 15:**
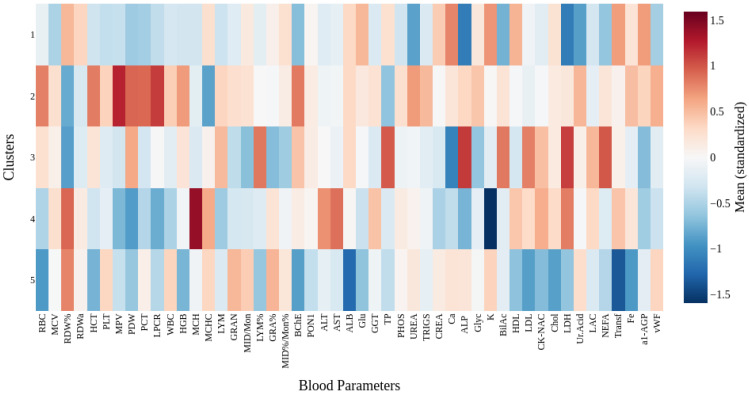
Heatmap of mean standardized blood parameters across clusters.

To further characterize the identified clusters, differences in mean blood parameter values were analyzed. Prior to the analysis, all features were standardized to ensure valid comparisons between variables with different scales. The resulting differences are visualized as a heatmap in Figure 15, illustrating the relative profiles of blood indicators for each cluster. This visualization highlights distinct trends in certain biochemical parameters, suggesting potential physiological distinctions among the identified groups.

To objectively assess the agreement between the model-estimated and statistical significance of features [6], a rank comparison was performed and the Spearman correlation was employed. The negative logarithm of the *p*-value from the nonparametric Kruskal-Wallis tests and Dunn’s post hoc test was used as a measure of the statistical significance of each feature: $-\log_{10}\left(p\right)$. The model-estimated significance of features was expressed as normalized mean absolute values:

$$w_{j}^{k}=E_{i\in G}\left[\left|\varphi_{ij}^{k}\right|\right],$$

$${\tilde{w}}_{j}^{k}=\frac{w_{j}^{k}}{\underset{j^{\prime }}{\max}{\;E}_{i\in G}\left[\left|\varphi_{ij^{\prime }}^{k}\right|\right]},$$

where $\varphi_{ij}^{k}$ are the SHAP values for feature (blood parameter) *j*, patient *i*, class *k*.

The Spearman correlation coefficient was calculated based on the ranks:

$$\rho =1-\;\frac{6\;\sum_{i}d_{i}^{2}}{n\;\left(n^{2}-1\right)},$$

where *d_i_* is the difference in ranks between the ranked sets of statistical significance (the negative logarithm of statistical significance) and the features selected as a result of the SHAP analysis; *n* is the number of features.

To determine the Spearman correlation coefficient, the sets of statistical significance of features and SHAP values were ranked—each element was assigned a rank $R_{ii^{\prime }}^{stat}$ and $R_{i}^{SHAP}$ according to the order in the set. The results of the comparative analysis are presented in Table 4 and Table 5.

### 2.7. External Validation on a Single-Class Cohort

To evaluate the generalization capability of the proposed framework and substantiate the clinical relevance of the identified biomarker signatures, external validation was performed on an independent, single-class cohort of patients diagnosed with SIVD. Given that the initial findings were considered exploratory due to the high-dimensional nature of the biochemical feature space (*p* ≫ *n*), this step aimed to determine whether the model-estimated feature importance remains stable when applied to a non-overlapping dataset.

The validation cohort comprised 28 patients, representing a clinical phenotype closely aligned with the SIVD group analyzed in the primary study. It should be noted that certain biomarkers, specifically {‘BilAc’, ‘LAC’, ‘Glyc’, ‘Transf’, ‘PON1’, ‘NEFA’, ‘Ur.Acid’, ‘a1-AGP’, ‘K’, ‘vWF’}, were unavailable in the validation dataset due to differences in clinical recording protocols. To maintain the integrity of the input feature vector and enable the deployment of the pre-trained classifier, these missing values were imputed using the mean values derived from the primary training dataset.

Furthermore, a rigorous comparative analysis was conducted between the primary and validation cohorts to assess potential out-of-distribution (OOD) effects and distributional shifts. This evaluation included mean values with 95% confidence intervals (CIs), medians, and interquartile ranges (IQRs) for each biochemical parameter. The Population Stability Index (PSI) was calculated for every feature; the average PSI was 2.111, indicating substantial distributional differences between the training and validation cohorts. These detailed comparative statistics and PSI values are provided in the Supplementary Materials (Tables S2 and S3).

**Figure 16:**
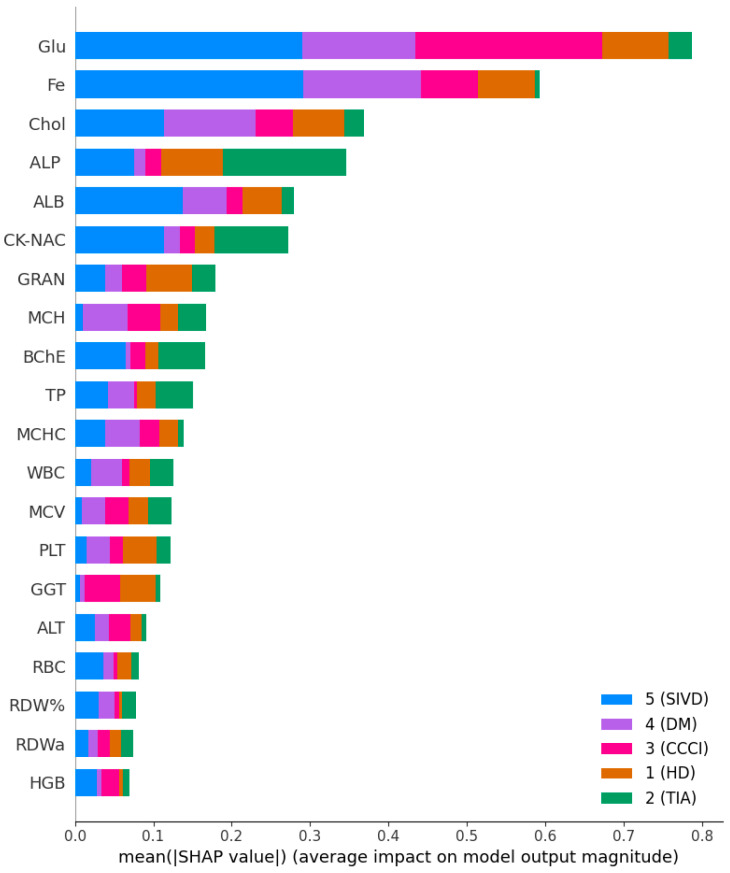
Mean absolute SHAP values for each feature, broken down by class, illustrating their contribution to the model predictions in the validation cohort.

To ensure methodological consistency and mitigate the impact of heteroscedasticity, the pre-trained Gradient Boosting model (maximum tree depth = 3) was deployed alongside the previously optimized variance-aware preprocessing pipeline with accuracy 71.43%. Performance was quantified not only by classification accuracy but also by the consistency of feature attribution, using SHAP (Figure 16) to assess whether key markers—such as iron (Fe), glucose, and transferrin—retain their synergistic predictive roles in an independent clinical setting. This external validation serves to confirm that the identified biomarker signatures are not artifacts of the original sample’s variance structure but reflect stable physiological patterns associated with vascular cognitive impairment.

## 3. Discussion

Classical classifiers (LDA, LR, kNN, DT, RF, SVM) were evaluated on three feature set variants: the full set, features selected via one-way ANOVA, and features selected via Welch’s ANOVA. The results revealed considerable variability across classes: for some classes, the models achieved acceptable accuracy, whereas for others, F1 scores were low (see Table 1). SHAP analysis and the interaction network identified a number of stable markers and inter-feature relationships (Figure 8, Figure 9, Figure 10, Figure 11, Figure 12 and Figure 13).

Several factors likely contribute to the observed limitations in classification performance. First, the dataset exhibits high dimensionality relative to the sample size, with approximately 49 numerical features for only 120 patients. This “wide” setup (*p* ≫ *n*) increases estimate variability, promotes overfitting, and destabilizes model parameters, particularly for linear models and SVMs with nonlinear kernels. Second, class imbalance shifts the optimization toward the more prevalent classes, reducing macro-F1 scores even when overall accuracy or dominant-class F1 appears satisfactory, as reflected in Table 1. Third, heterogeneity within clinical groups, arising from diverse comorbidities, increases within-class variance and diminishes between-class discriminability. This is consistent with high heteroscedasticity observed in several features (Levene test), explaining differences in feature rankings between Welch’s and standard ANOVA. Finally, strong correlations and redundancy among features (e.g., RBC/HGB/HCT; PLT/PCT; Chol/LDL) complicate coefficient interpretation and dilute the contribution of individual variables in linear models.

SHAP analysis further provided insights into feature importance. A consistent set of markers—including Fe, Transferrin, RDW%, LDL/Chol, LDH, vWF, Glu, ALB, and several hematological parameters—was repeatedly identified across classes. These features not only show high individual contributions but also participate in synergistic interactions, as evidenced in the SHAP interaction network (Figure 8, Figure 9, Figure 10, Figure 11, Figure 12 and Figure 13). Importantly, feature relevance is class-specific: certain markers are critical for distinguishing vascular-metabolic conditions, while others are more relevant for cognitive-vascular syndromes. This class-specificity explains why global metrics, such as macro-F1, may not fully capture the clinical utility of the models for particular diagnoses.

The results of the comparative analysis of ranks showed that there is a consistency between the results of the statistical analysis and the SHAP method—it is especially pronounced for combinations of groups 1–2 (0.5277) and 1–5 (0.5919) according to the Spearman coefficient. However, blood parameters that are important for predicting groups, but not for statistical analysis, were also identified: for group 2, they were Glu, Transf, LAC, WBC, LDL; for group 3, they were UREA, Ca, CREA, Fe, BilAc; for group 4, they were Chol, Fe, HDL, Ur. Acid, Ca; and for group 5, they were MCHC, Ca, K-RUV, BilAc, NEFA.

To further assess model robustness, all analyses were validated on an independent cohort consisting exclusively of patients with dementia. Statistical comparisons between the original and dementia-specific cohorts revealed a significant shift in data distributions, and several features present in the original dataset were unavailable in the new cohort. Despite these limitations, the gradient boosting model, combined with SHAP-based interpretability, maintained high classification accuracy. Notably, the same set of stable markers and class-specific feature interactions identified in the original cohort were largely preserved, indicating that the predictive patterns uncovered are robust to cohort heterogeneity and missing variables, at least within the context of dementia patients.

## 4. Materials and Methods

### 4.1. Chemicals

PBS (pH 7.4) was purchased from Biolot (St. Petersburg, Russia). Diagnostic kits were produced by Randox Laboratories (Crumlin, UK). All other reagents were from Sigma-Aldrich (Rockville, MD, USA).

### 4.2. Study Design and Setting

The study was conducted in accordance with the Declaration of Helsinki and approved by the Ethics Committee of the Research Institute of Hygiene, Occupational Pathology and Human Ecology of the Federal Medical Biological Agency (Approval No. 3, registration date: 2 June 2022). Informed consent was obtained from all subjects involved in this study. The period during which the patient groups were formed, and their neurological examination and blood sampling for subsequent analysis were performed was approximately one year—from mid-2022 to mid-2023. The dataset comprised 120 elderly patients (60 to 78 years old) living in St. Petersburg and registered in the geriatric departments of city clinics. Structured interviews were conducted to obtain socio-demographic data. Medical history and neurological examination data were collected. The patients were divided into five groups based on clinical diagnoses established according to standard criteria detailed in the prior study [6]. Exclusion criteria included data on progressive cancer and recent infectious diseases. As for the inclusion criteria, numerical labels (1–5) map to the following groups for consistency across analyses (Table 6). For validation purposes, a group of 28 patients aged 60–80 undergoing inpatient treatment at City Hospital No. 28 in St. Petersburg was formed in the fall of 2025.

### 4.3. Sample Preparation

Blood samples were collected, processed, and stored in accordance with international guidelines [32]. Blood was collected from the subjects on an empty stomach from the cubital vein into BD Vacutainer vacuum tubes, Becton Dickinson, Franklin Lakes, NJ, USA with anticoagulants (K3EDTA, heparin, and citrate). The plasma was stored at −70 °C until needed.

### 4.4. Biochemical and Hematological Analysis

Biochemical parameters were determined using a Sapphire 400 analyzer (Tokyo Boeki Medisys, Tokyo, Japan) and commercial RANDOX kits. Immunological and hematological studies of whole blood were performed on the day of blood collection. Baseline parameters and histograms of the distribution of erythrocytes, leukocytes, and platelets by volume were determined using a Medonic hematology analyzer (Boule Diagnostics, Spånga, Sweden).

### 4.5. Von Willebrand Factor and ADAMTS13

To quantify vWF in blood plasma using the standard method, the Technozym vWF:Ag ELISA kit (Technoclone GmbH, Vienna, Austria) was used. The activity of the von Willebrand factor in the plasma of patients was determined in the agglutination reaction of lyophilized platelets with ristocetin using a set of reagents from the Renam company (Moscow, Russia, No. AG-5). The determination of ADAMTS13 activity (VWF cleaving agent) in plasma was carried out using a Synergy 2 plate fluorometer (BioTech, Hudson, MA, USA) and the fluorescent substrate FRETS-VWF73.

### 4.6. Dataset Description

This study utilized a dataset comprising biochemical blood parameters from 120 patients divided into four groups: control (group 1), AIS (group 2), CCCI (group 3), DM (group 4) and SIVD (group 5). The dataset included 49 features such as RBC, MCV, PLT, and various enzymes (e.g., BChE, TP, vWF). In Table 7 below is the detail description of the dataset. Data preprocessing involved imputation of missing values using median filling, normalization via StandardScaler to ensure zero mean and unit variance, and outlier detection with a contamination level of 0.01 to reduce noise in heterogeneous biomedical data. All analyses were conducted using Python 3.10 with scikit-learn (v. 1.8.0), NumPy (v. 2.3.5), Pandas (v. 2.3.3), SHAP (v. 0.48.0), CatBoost (v. 1.2.8) and Matplotlib (v. 3.10.8) libraries.

#### 4.6.1. Feature Selection Methods

To address heteroscedasticity and enhance model robustness, three feature selection methods were applied, with a specific focus on mitigating the risks associated with violated statistical assumptions.

- Full Feature Set: All 49 blood parameters were used without filtering, serving as a baseline to assess the impact of the complete biomarker set on classification performance. This “wide” setup (*p* ≫ *n*) often increases estimate variability and promotes overfitting in high-dimensional biomedical data, necessitating comparative selection strategies.
- One-Way ANOVA Method: Features were selected based on standard ANOVA, which evaluates differences between groups by comparing mean values. While this method assumes homogeneity of variances (homoscedasticity), it was employed as a comparative benchmark to identify biomarkers with significant variance across imbalanced groups. However, when this assumption is violated, the standard F-test becomes unreliable, leading to Type I error rates that deviate substantially from nominal significance levels and potentially producing spurious results.
- Welch’s ANOVA Method (Variance-Aware): To account for the unequal variances typical in clinical biochemical data, Welch’s ANOVA was applied. This method is specifically designed to handle heteroscedasticity by adjusting the F-statistic and degrees of freedom

The justification for this approach is twofold:

(1) Empirical Evidence: In our study, Levene’s test confirmed statistically significant heterogeneity of variance (*p* < 0.05) for several critical features, including iron (Fe), transferrin (Transf), RDW%, and LDL receptor (LDL). These findings directly demonstrated that the equal-variance assumption was untenable for our dataset.
(2) Methodological Robustness: Welch’s ANOVA provides more accurate Type I error control and maintains comparable or superior statistical power to classical ANOVA even when variances are equal. It effectively eliminates the need for homogeneity as a prerequisite, ensuring that the identified biomarker signatures are not artifacts of unequal group variances.

Features were ranked according to their F-statistics, and the comparison of *p*-values between the two ANOVA variants revealed marked discrepancies in statistical significance for key parameters, underscoring the necessity of using variance-aware workflows to ensure the reliability of subsequent machine learning tasks.

#### 4.6.2. ML Models

Six classical supervised classification algorithms were employed to predict patient group membership based on the selected blood biomarkers. Prior to model training, feature values were standardized to ensure consistent scaling across variables. The chosen models represent a complementary spectrum of analytical approaches: linear methods facilitate interpretability and identification of direct associations, whereas nonlinear models are capable of capturing intricate, nonlinearly separable patterns inherent in heterogeneous clinical datasets.

##### Linear Discriminant Analysis (LDA)

LDA is a classical statistical approach that projects data onto a lower-dimensional space to achieve maximum class separability. It constructs a linear combination of features that optimally distinguishes between predefined groups by maximizing the ratio of between-class to within-class variance [33].

##### Logistic Regression (LR)

LR was employed as a complementary linear classifier to benchmark model performance. It models the probability of class membership using the logistic (sigmoid) function and provides interpretable coefficients that quantify each feature’s contribution to the prediction. In this study, LR was extended to multiclass classification using the one-vs-rest strategy. To mitigate overfitting and improve generalization, both L1 (Lasso) and L2 (Ridge) regularization techniques were applied [34].

##### k-Nearest Neighbor (kNN)

KNN is a non-parametric, instance-based learning algorithm that classifies samples based on their similarity to previously observed data. Rather than building an explicit model during training, KNN stores all training examples and determines the class of a new sample by analyzing the labels of its closest neighbors in the feature space. kNN captured local patterns in the biochemical feature space [35].

##### Decision Tree (DT)

A non-linear, tree-based method that recursively partitions the feature space based on feature thresholds to minimize impurity. DT was included to model potential non-linear decision boundaries [36,37].

##### Random Forest (RF)

An ensemble of decision trees that aggregates predictions via majority voting, offering robustness to overfitting and high-dimensional data. RF was used here to handle feature redundancy and interactions among correlated biochemical markers [38]. Key parameters tuned during hyperparameter optimization include:

N estimators: This defines the number of decision trees in the RF. Values 50, 100 and 200 were tested to strike a balance between predictive performance and computational efficiency.

Max_depth: This controls the maximum number of splits per decision tree. A low value risks underfitting, while a high value may lead to overfitting. In this study, tree depths were adjusted to 5, 10 and 15 to capture meaningful patterns without excessive complexity.

These parameter adjustments resulted in a robust and accurate model, well-suited for handling the complexity and high dimensionality of biological data.

##### Support Vector Machines (SVM)

A kernel-based method that finds an optimal hyperplane, maximizing the margin between classes, particularly effective for high-dimensional data. SVM was applied to capture potentially non-linear separations in the biomarker space; tuned parameters included regularization parameter C, kernel type, and gamma parameter for RBF and polynomial kernels [39].

#### 4.6.3. Cross-Validation

To ensure robust model evaluation and prevent overfitting, stratified k-fold cross-validation was employed with *k* = 3. Stratification preserved the class distribution within each fold, reflecting the multiclass nature of the dataset (four patient groups). The dataset was partitioned into non-overlapping training and validation subsets for each iteration, with models trained on the training set and evaluated on the test set. This process was repeated across all folds, and performance metrics were averaged to improve reliability and assess model sensitivity to training subsets.

#### 4.6.4. Hyperparameter Optimization

Hyperparameters were tuned using GridSearchCV, an exhaustive search over a predefined parameter grid with internal 3-fold cross-validation, using the macro-averaged F1-score as the evaluation metric. The tuning parameters for each model included:

LDA: Solver type (‘svd’, ‘lsqr’, ‘eigen’) and shrinkage parameter (applicable for ‘lsqr’ and ‘eigen’ solvers). In this study, the default ‘svd’ solver was used without additional shrinkage optimization, as it is robust to multicollinearity and does not require regularization tuning.

Logistic Regression (LR): Regularization type (‘l1’, ‘l2’, ‘elasticnet’, ‘none’), regularization strength (C ∈ {0.01, 0.1, 1, 10, 100}), solver (‘saga’, compatible with all penalty types), and L1 ratio (∈{0, 0.5, 1}, applicable only for elastic-net regularization). Based on GridSearchCV results, the optimal LR configuration employed L1 regularization with *C* = 1, using the saga solver.

K-Nearest Neighbors (KNN): Number of neighbors (k ∈ {3, 5, 7, 9, 11}), weighting strategy (‘uniform’, ‘distance’), and distance metric (‘euclidean’, ‘manhattan’). Based on GridSearchCV results, the optimal KNN configuration employed *k* = 9 neighbors, distance-based weighting, and the Euclidean distance metric.

Decision Tree (DT): Maximum tree depth (None, 3, 5, 7, 9), minimum number of samples required to split an internal node (2, 5, 10), minimum number of samples required at a leaf node (1, 2, 4), and splitting criterion (‘gini’, ‘entropy’). Based on GridSearchCV results, the optimal DT configuration employed a maximum tree depth of 5, a minimum of 2 samples per leaf, a minimum of 2 samples to split an internal node, and the entropy-based splitting criterion.

Random Forest (RF): Number of trees (n_estimators ∈ {50, 100, 200}), maximum tree depth (None, 5, 10, 15), minimum number of samples required to split an internal node (2, 5, 10), minimum number of samples required at a leaf node (1, 2, 4), and splitting criterion (‘gini’, ‘entropy’). Based on GridSearchCV results, the optimal RF configuration employed 50 trees, no explicit limit on tree depth, a minimum of 5 samples to split an internal node, a minimum of 1 sample per leaf, and the entropy-based splitting criterion.

Support Vector Machine (SVM): Regularization parameter C (0.1, 1, 10, 100), kernel type (‘linear’, ‘rbf’, ‘poly’), and kernel coefficient gamma (‘scale’, ‘auto’; used only for ‘rbf’ and ‘poly’ kernels). Based on GridSearchCV results, the optimal SVM configuration employed an RBF kernel with *C* = 10 and *γ* = scale.

Optimal hyperparameters were selected based on the highest cross-validated F1-score to ensure strong generalization capability on unseen data.

#### 4.6.5. Evaluation Metrics

Model performance was assessed using the macro-averaged F1-score, balancing precision and recall across all classes, appropriate for multiclass, imbalanced datasets.

Mean F1-scores and standard deviations were calculated across cross-validation folds for each pipeline (full feature set, one-way ANOVA, Welch’s ANOVA).

Ethical considerations complied with institutional guidelines, and data were anonymized to protect patient confidentiality [40].

## 5. Conclusions

In this study, a variance-robust and interpretable analytical framework was proposed and validated for biomarker discovery in multivariate clinical datasets characterized by small sample sizes, heteroscedasticity, and overlapping diagnostic categories. It was demonstrated that ignoring variance heterogeneity leads to unstable feature selection and potentially misleading interpretations, while the use of Welch’s ANOVA provides more robust results without sacrificing statistical power.

Integrating interpretable machine learning methods with SHAP analysis enables the identification of nonlinear, class-specific, and synergistic effects between biochemical markers that go beyond traditional single-variate statistics. The results demonstrate that interpretability is not simply an auxiliary tool for explaining patterns, but a critical component of robust clinical machine learning.

The proposed workflow can be directly applied to other biomedical fields where data heterogeneity and limited sample sizes make robust statistical inference difficult, providing a transparent link between statistical testing, predictive modeling, and clinical interpretation.

## 6. Limitations of the Research

Several limitations of the present study should be acknowledged. First, the analysis is based on a relatively small cohort (*n* = 120) while the feature space has high dimensionality (≈49 biochemical parameters), resulting in a *p* ≫ *n* configuration that increases estimator variability and the risk of overfitting despite cross-validation and model regularization. This limited sample size reduces the statistical power to detect small-to-moderate effects and constrains the generalizability of the findings.

Second, the dataset contains substantial inter-feature correlation and redundancy (e.g., RBC/HGB/HCT, PLT/PCT, Chol/LDL), which complicates model interpretability and can destabilize feature importance rankings; although SHAP provides local and global explainability, correlated predictors may still lead to ambiguous attribution of effects.

Third, several parameters exhibit pronounced heteroscedasticity (Levene’s test), and one of the groups shows marked separation in the clustering analysis. Unequal variances across groups violate assumptions of classical parametric tests and can bias significance rankings and cross-group comparisons; while Welch’s ANOVA and variance-aware procedures were employed to mitigate this issue, residual effects of heteroscedasticity and group-specific variance structure may still affect both statistical and machine-learning inferences.

Finally, class imbalance and the moderate agreement between unsupervised clusters and clinical labels (low ARI/NMI and modest Silhouette scores) limit the immediate clinical translatability of the classifiers. Together, these limitations mean that the identified biomarker signatures should be considered exploratory; future work must validate the results on larger, multi-center cohorts, apply more extensive external validation, and test robustness to alternative preprocessing, dimensionality-reduction, and class-balancing strategies.

## Figures and Tables

**Table 1:** F1 Metrics by Class for Various Classification Models.

Model	Data Representation	Class 1 (HD)	Class 2 (TIA)	Class 3 (CCCI)	Class 4 (DM)	Class 5 (SIVD)	Macro-F1
LDA	Full	0.37 ± 0.06	0.23 ± 0.09	0.18 ± 0.05	0.44 ± 0.23	0.74 ± 0.02	0.39
	One-way	0.36 ± 0.15	0.35 ± 0.08	0.30 ± 0.09	0.38 ± 0.04	0.86 ± 0.10	0.45
	Welch	0.30 ± 0.04	0.36 ± 0.09	0.38 ± 0.14	0.37 ± 0.03	0.78 ± 0.06	0.44
kNN	Full	0.48 ± 0.10	0.33 ± 0.08	0.19 ± 0.03	0.47 ± 0.14	0.80 ± 0.04	0.45
	One-way	0.55 ± 0.04	0.46 ± 0.11	0.18 ± 0.13	0.49 ± 0.13	0.89 ± 0.14	0.51
	Welch	0.54 ± 0.01	0.43 ± 0.03	0.18 ± 0.13	0.54 ± 0.13	0.91 ± 0.08	0.52
LR	Full	0.45 ± 0.07	0.43 ± 0.17	0.30 ± 0.09	0.39 ± 0.07	0.83 ± 0.02	0.48
	One-way	0.53 ± 0.14	0.24 ± 0.17	0.37 ± 0.15	0.42 ± 0.04	0.72 ± 0.16	0.46
	Welch	0.51 ± 0.14	0.38 ± 0.07	0.38 ± 0.16	0.41 ± 0.06	0.83 ± 0.04	0.50
DT	Full	0.36 ± 0.19	0.27 ± 0.06	0.26 ± 0.09	0.32 ± 0.08	0.58 ± 0.14	0.36
	One-way	0.43 ± 0.19	0.31 ± 0.09	0.30 ± 0.05	0.34 ± 0.19	0.58 ± 0.06	0.39
	Welch	0.37 ± 0.06	0.28 ± 0.07	0.36 ± 0.08	0.36 ± 0.13	0.67 ± 0.13	0.41
RF	Full	0.47 ± 0.12	0.47 ± 0.07	0.26 ± 0.12	0.54 ± 0.09	0.82 ± 0.1	0.51
	One-way	0.50 ± 0.12	0.44 ± 0.09	0.23 ± 0.16	0.49 ± 0.04	0.82 ± 0.15	0.50
	Welch	0.55 ± 0.13	0.43 ± 0.11	0.34 ± 0.18	0.52 ± 0.03	0.84 ± 0.08	0.54
SVM	Full	0.63 ± 0.03	0.37 ± 0.13	0.31 ± 0.17	0.48 ± 0.11	0.81 ± 0.11	0.52
	One-way	0.55 ± 0.14	0.29 ± 0.07	0.14 ± 0.09	0.37 ± 0.11	0.72 ± 0.04	0.41
	Welch	0.54 ± 0.17	0.35 ± 0.03	0.19 ± 0.13	0.41 ± 0.15	0.72 ± 0.11	0.44

**Table 2:** Performance metrics of the Gradient Boosting model for each class.

Class	F1-Score	Precision	Recall
Class 1 (HD)	0.96	0.96	0.86
Class 2 (TIA)	0.89	0.88	0.76
Class 3 (CCCI)	0.87	1.00	0.91
Class 4 (DM)	0.94	0.91	0.81
Class 5 (SIVD)	0.95	0.91	0.81

**Table 3:** Composition of clusters with respect to ground-truth classes. The share of the dominant class in the entire cluster is shown in parentheses.

Class	Cluster Label				
	Cluster 1	Cluster 2	Cluster 3	Cluster 4	Cluster 5
Class 1	7	4	5	6	1
Class 2	1	7	12	3	0
Class 3	6	7	3	5	1
Class 4	13	12	5	1	1
Class 5	1	0	0	0	19
Dominant	DM—4 (0.46)	DM—4 (0.40)	TIA—2 (0.48)	HD—1 (0.40)	SIVD—5 (0.86)

**Table 4:** Significance of Differences Across Groups Relative to the Reference Group.

Group Combination	ρ
1–2	0.5277
1–3	0.4849
1–4	0.2492
1–5	0.5919

**Table 5:** Comparison of Feature Rankings Based on Spearman Correlation and SHAP Values.

Feature Name	$d^{12}$	${R_{12}}^{stat}$	${R_{2}}^{SHAP}$	$d^{13}$	${R_{13}}^{stat}$	${R_{3}}^{SHAP}$	$d^{14}$	${R_{14}}^{stat}$	${R_{4}}^{SHAP}$	$d^{15}$	${R_{15}}^{stat}$	${R_{5}}^{SHAP}$
RBC	3.0	18.0	15.0	9.0	13.0	4.0	−5.0	5.0	10.0	13.0	41.0	28.0
MCV	8.0	37.0	29.0	1.0	39.0	38.0	−17.0	16.0	33.0	4.0	11.0	7.0
RDW%	4.0	32.0	28.0	−17.0	3.0	20.0	3.0	30.0	27.0	−15.0	23.0	38.0
RDWa	4.0	35.0	31.0	−18.0	9.0	27.0	15.0	34.0	19.0	−4.0	27.0	31.0
HCT	2.0	29.0	27.0	−1.0	23.0	24.0	−1.0	14.0	15.0	8.0	34.0	26.0
PLT	−14.0	11.0	25.0	9.0	37.0	28.0	9.0	40.0	31.0	13.0	30.0	17.0
MPV	8.0	14.0	6.0	3.0	8.0	5.0	8.0	17.0	9.0	0.0	5.0	5.0
PDW	7.0	16.0	9.0	−5.0	2.0	7.0	0.0	8.0	8.0	7.0	22.0	15.0
PCT	11.0	12.0	1.0	32.0	33.0	1.0	37.0	44.0	7.0	24.0	25.0	1.0
LPCR	−10.0	8.0	18.0	−4.0	7.0	11.0	−12.0	9.0	21.0	−10.0	19.0	29.0
WBC	−19.0	17.0	36.0	12.0	31.0	19.0	−1.0	38.0	39.0	16.0	37.0	21.0
HGB	12.0	28.0	16.0	−8.0	21.0	29.0	−2.0	12.0	14.0	16.0	38.0	22.0
MCH	−10.0	33.0	43.0	−13.0	35.0	48.0	−24.0	22.0	46.0	−4.0	9.0	13.0
MCHC	33.0	41.0	8.0	10.0	42.0	32.0	8.0	49.0	41.0	−33.0	1.0	34.0
LYM	3.0	5.0	2.0	9.0	11.0	2.0	1.0	2.0	1.0	8.0	10.0	2.0
GRAN	−12.0	21.0	33.0	8.0	43.0	35.0	16.0	48.0	32.0	7.0	40.0	33.0
MID/Mon	−2.0	2.0	4.0	12.0	15.0	3.0	27.0	29.0	2.0	15.0	18.0	3.0
LYM%	−12.0	10.0	22.0	21.0	44.0	23.0	14.0	43.0	29.0	19.0	35.0	16.0
GRA%	−17.0	13.0	30.0	8.0	45.0	37.0	3.0	45.0	42.0	9.0	36.0	27.0
MID%/Mon%	16.0	23.0	7.0	20.0	26.0	6.0	16.0	20.0	4.0	3.0	13.0	10.0
BChE	4.0	43.0	39.0	0.0	22.0	22.0	2.0	19.0	17.0	−1.0	42.0	43.0
PON1	14.0	26.0	12.0	16.0	25.0	9.0	17.0	37.0	20.0	12.0	26.0	14.0
ALT	−10.0	4.0	14.0	−1.0	29.0	30.0	9.0	27.0	18.0	−4.0	14.0	18.0
AST	31.0	34.0	3.0	14.0	28.0	14.0	28.0	39.0	11.0	1.0	12.0	11.0
ALB	2.0	19.0	17.0	−5.0	16.0	21.0	7.0	31.0	24.0	3.0	45.0	42.0
Glu	−33.0	9.0	42.0	0.0	49.0	49.0	−5.0	42.0	47.0	−1.0	46.0	47.0
GGT	14.0	24.0	10.0	2.0	46.0	44.0	19.0	32.0	13.0	9.0	15.0	6.0
TP	−4.0	36.0	40.0	2.0	19.0	17.0	4.0	41.0	37.0	−3.0	33.0	36.0
PHOS	−10.0	3.0	13.0	−4.0	12.0	16.0	0.0	23.0	23.0	−1.0	8.0	9.0
UREA	1.0	42.0	41.0	−37.0	5.0	42.0	−13.0	25.0	38.0	−5.0	7.0	12.0
TRIGS	2.0	7.0	5.0	24.0	34.0	10.0	18.0	21.0	3.0	2.0	6.0	4.0
CREA	−2.0	30.0	32.0	−30.0	4.0	34.0	−21.0	15.0	36.0	−15.0	17.0	32.0
Ca	−2.0	45.0	47.0	−31.0	10.0	41.0	−27.0	1.0	28.0	−26.0	4.0	30.0
ALP	0.0	48.0	48.0	−11.0	20.0	31.0	17.0	33.0	16.0	−15.0	24.0	39.0
Glyc	8.0	27.0	19.0	0.0	40.0	40.0	10.0	35.0	25.0	−9.0	28.0	37.0
K	−9.0	25.0	34.0	−12.0	1.0	13.0	−24.0	6.0	30.0	−25.0	16.0	41.0
BilAc	17.0	38.0	21.0	−19.0	6.0	25.0	−12.0	10.0	22.0	−22.0	3.0	25.0
HDL	29.0	40.0	11.0	3.0	36.0	33.0	−30.0	4.0	34.0	4.0	39.0	35.0
LDL	−18.0	6.0	24.0	17.0	32.0	15.0	1.0	13.0	12.0	24.0	48.0	24.0
CK-NAC	−15.0	31.0	46.0	−2.0	24.0	26.0	2.0	28.0	26.0	−13.0	32.0	45.0
Chol	1.0	39.0	38.0	−6.0	41.0	47.0	−46.0	3.0	49.0	1.0	47.0	46.0
LDH	0.0	44.0	44.0	2.0	14.0	12.0	5.0	11.0	6.0	−15.0	29.0	44.0
Ur.Acid	1.0	46.0	45.0	1.0	47.0	46.0	−27.0	18.0	45.0	11.0	31.0	20.0
LAC	−22.0	1.0	23.0	9.0	17.0	8.0	1.0	36.0	35.0	13.0	21.0	8.0
NEFA	12.0	47.0	35.0	12.0	30.0	18.0	19.0	24.0	5.0	−17.0	2.0	19.0
Transf	−27.0	22.0	49.0	−16.0	27.0	43.0	−14.0	26.0	40.0	0.0	49.0	49.0
Fe	−5.0	15.0	20.0	−27.0	18.0	45.0	−41.0	7.0	48.0	−4.0	44.0	48.0
a1-AGP	−6.0	20.0	26.0	2.0	38.0	36.0	2.0	46.0	44.0	−3.0	20.0	23.0
vWF	12.0	49.0	37.0	9.0	48.0	39.0	4.0	47.0	43.0	3.0	43.0	40.0

**Table 6:** Patient groups and inclusion criteria.

Label	Class	Diagnostic Criteria	*n*
1	HD (healthy donors)	No history of acute or chronic cerebrovascular accident, signs of diabetes or cognitive impairment.	23
2	AIS (acute ischemic stroke)	At least one registered visit to the clinic in the last 3 years due to acute cerebrovascular accident, confirmed by instrumental analysis data.	24
3	CCCI (chronic cerebral circulation insufficiency)	Neurological examination and instrumental analysis data indicate the presence of chronic cerebral circulation insufficiency.	21
4	DM (type 2 diabetes)	Clinical and biochemical features of early and untreated type 2 diabetes.	32
5	SIVD (subcortical ischemic vascular dementia)	Neurological examination and instrumental analysis data indicate the presence of subcortical ischemic vascular dementia.	20

**Table 7:** Description of features in the dataset, including feature names, detailed descriptions, and units of measurement.

Feature Name	Description	Units
Patient group	Disease class (1 = HD, 2 = TIA, 3 = CCH, 4 = DM, 5 = SIVD)	
Gender	1= male, 0= female	
RBC	red blood cells	10^6^/μL
MCV	mean corpuscular volume	fL
RDW%	red cell distribution width (relative value)	%
RDWa	red cell distribution width (absolute value)	fL
HCT	hematocrit	%
PLT	platelets	10^3^/μL
MPV	mean platelet volume	fL
PDW	platelet distribution width by volume	%
PCT	plateletcrit	%
LPCR	large platelet coefficient	%
WBC	white blood cells	10^3^/μL
HGB	hemoglobin	g/dL
MCH	mean corpuscular hemoglobin	pg
MCHC	mean corpuscular hemoglobin concentration	g/dL
LYM	lymphocytes (absolute)	10^3^/μL
GRAN	granulocytes (absolute)	10^3^/μL
MID/Mon	monocytes/medium cells (absolute)	10^3^/μL
LYM%	lymphocytes	%
GRA%	granulocytes	%
MID%/Mon%	monocytes/medium cells	%
BChE	butyrylcholinesterase	U/L
PON1	paraoxonase 1 (enzyme)	U/L
ALT	alanine aminotransferase	U/L
AST	aspartate aminotransferase	U/L
ALB	albumin	g/L
Glu	glucose	mmol/L
GGT	gamma-glutamyl transferase	U/L
Tot.Prot	total protein	g/L
PHOS	inorganic phosphorus	mmol/L
UREA	urea	mmol/L
TRIGS	triglycerides	mmol/L
CREA	creatinine	μmol/L
ALP	alkaline phosphatase	U/L
K	potassium (measurement method)	mmol/L
BilAc	direct (conjugated) bilirubin	μmol/L
HDL	high-density lipoprotein cholesterol	mmol/L
LDL	low-density lipoprotein cholesterol	mmol/L
CK-NAC	creatine kinase	U/L
Chol	total cholesterol	mmol/L
LDH	lactate dehydrogenase	U/L
Ur.Acid	uric acid	μmol/L
LAC	lactate	mmol/L
NEFA	non-esterified fatty acids	mmol/L
Transf	transferrin	g/L
Fe	iron	μmol/L
a1-AGP	alpha-1-acid glycoprotein	g/L

## Data Availability

The original contributions presented in this study are included in the article. Further inquiries can be directed to the corresponding author.

## Supplementary Materials

The following supporting information can be downloaded at: https://www.mdpi.com/article/10.3390/ijms27041805/s1.

## Abbreviations

a1-AGP	Alpha-1-acid glycoprotein
ADAMTS13	A Disintegrin And Metalloproteinase with a ThromboSpondin type 1 motif, member 13
AI	Artificial Intelligence
AIS	Acute ischemic stroke
ALB	Albumin
ALP	Alkaline phosphatase
ALT	Alanine aminotransferase
ANCOVA	Analysis of covariance
ANOVA	Analysis of variance
ARI	Adjusted Rand Index
AST	Aspartate aminotransferase
BilAc	Direct bilirubin
BChE	Butyrylcholinesterase
BMI	Body mass index
CCCI	Chronic cerebral circulation insufficiency
Chol	Total cholesterol
CI	Confidence interval
CK-NAC	Creatine kinase
CREA	Creatinine
DM	Type 2 diabetes mellitus
DT	Decision Tree
ERD	Early dementia risk
Fe	Iron
Gender	Gender
GGT	Gamma-glutamyl transferase
Glu	Glucose
GRA%	Granulocyte percentage
GRAN	Granulocyte absolute count
HC3	Heteroskedasticity-Consistent Covariance Matrix Estimator
HCT	Hematocrit
HD	Healthy donor
HDL	High-density lipoprotein cholesterol
HGB	Hemoglobin
IQR	Interquartile range
K	Potassium
kNN	k-Nearest Neighbors
LAC	Lactate
LDA	Linear Discriminant Analysis
LDH	Lactate dehydrogenase
LDL	Low-density lipoprotein cholesterol
LPCR	Large platelet ratio
LR	Logistic Regression
LYM	Lymphocyte absolute count
LYM%	Lymphocyte percentage
MCH	Mean corpuscular hemoglobin
MCHC	Mean corpuscular hemoglobin concentration
MCV	Mean corpuscular volume
MID%/Mon%	Monocyte percentage
MID/Mon	Monocyte absolute count
MMSE	Mini-mental state examination
ML	Machine Learning
MOCA	Montreal cognitive assessment
MPV	Mean platelet volume
MVD	Microvascular dysfunction
NEFA	Non-esterified fatty acids
NMI	Normalized Mutual Information
PCT	Plateletcrit
PDW	Platelet distribution width
PHOS	Inorganic phosphorus
PLT	Platelets
PON1	Paraoxonase 1
PSI	Population Stability Index
RBC	Red blood cells
RDW	Red cell distribution width
RDWa	Red cell distribution width absolute
RF	Random Forest
Set Cover	Set cover algorithm
SIVD	Subcortical ischemic vascular dementia
SVM	Support Vector Machine
TIA	Transient ischemic attack
Tot. Prot., TP	Total protein
Transf	Transferrin
TRIGS	Triglycerides
Uric Acid	Uric acid
UREA	Urea
VWF	Von Willebrand factor
WBC	White blood cells
WCSS	Within-cluster sum of squares
