# Supplementary material for: Interpretable Machine Learning with SHAP Identifies Key Biomarkers in a Multi-Factorial Spectrum of Age-Related Neurological and Metabolic Conditions
Source: Int J Mol Sci. 2026 Feb 13;27(4):1805. doi: 10.3390/ijms27041805 (PMC12941188; doi:10.3390/ijms27041805)
Supplement: Supplementary file 1 [file ijms-27-01805-s001.zip › ijms-4110826-supplementary.pdf]

Supplementary materials

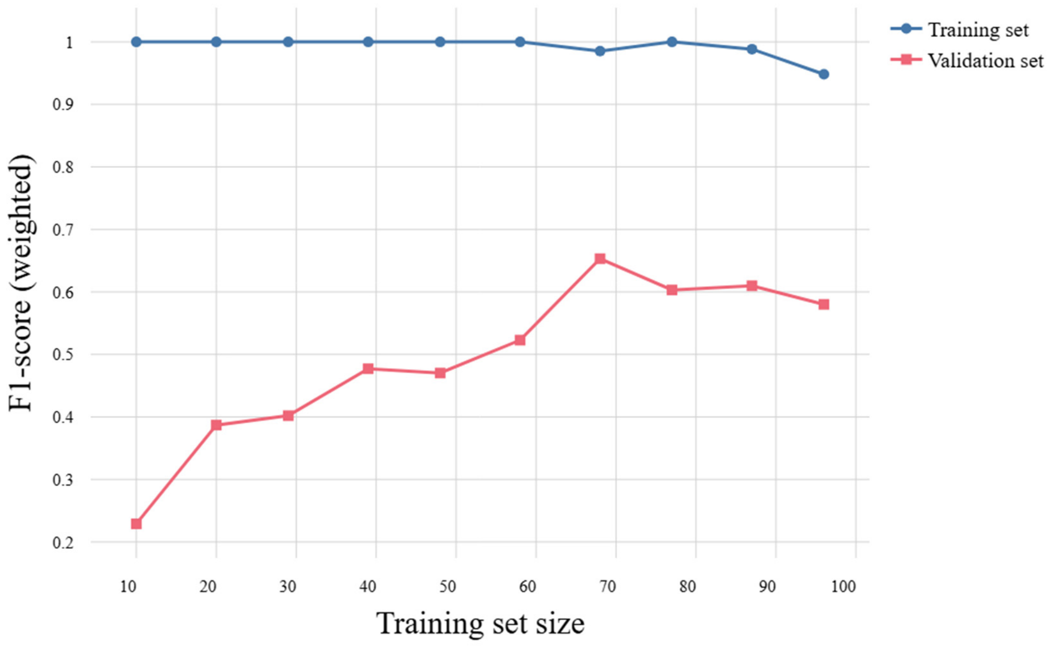

Figure S1. Learning Curves for the Gradient Boosting Classifier.

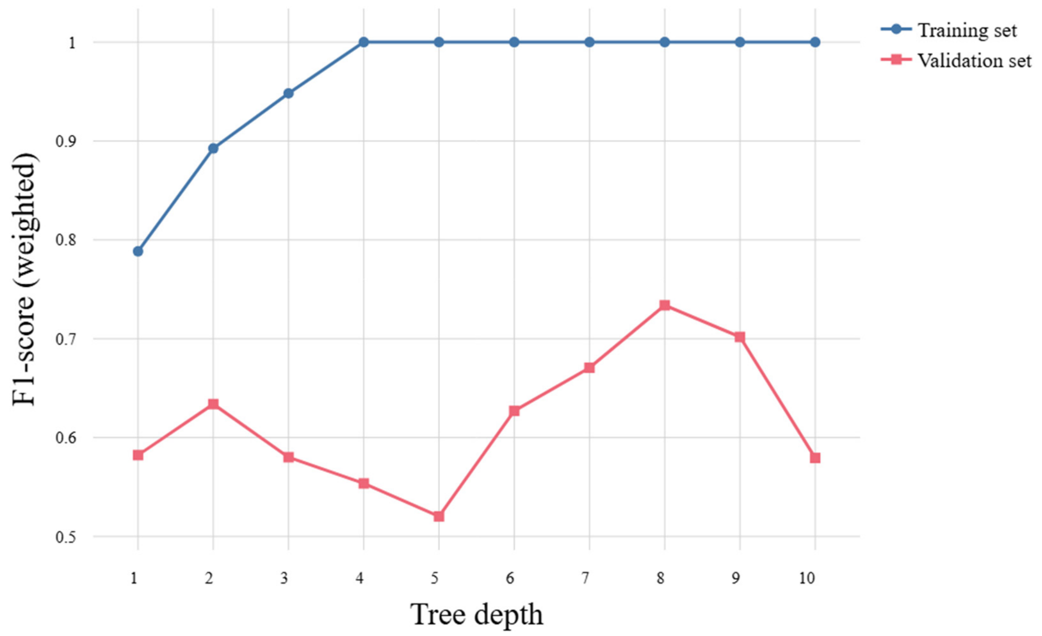

Figure S2. Sensitivity Analysis of Model Performance to Tree Depth.

Supplementary Table S1. Quantitative comparison of ANOVA variants demonstrates heteroscedasticity-induced inference distortion and Welch ANOVA consensus with robust alternatives (Kruskal–Wallis, Brown–Forsythe, HC3).

Feature name	ANOVA, p	Welch's ANOVA, p	Levene test, p	Brown-Forsythe, p	HC3, p	Kruskal–Wallis, p
RBC	8,5051E-04	6,6999E-04	4,5700E-02	4,5615E-02	8,3009E-04	1,1782E-03
MCV	3,5927E-02	1,2066E-01	7,1900E-02	8,3296E-02	3,8011E-02	1,0253E-01
RDW%	9,0747E-04	1,2655E-06	1,3000E-03	4,8300E-03	7,0234E-04	3,8529E-03
RDWa	7,9190E-01	5,2503E-03	5,8950E-01	5,7400E-04	1,0486E-04	2,8844E-04
HCT	3,3307E-03	4,0513E-03	3,3000E-02	3,3975E-02	3,4884E-03	4,2483E-03
PLT	7,1862E-02	1,0372E-01	6,6810E-01	6,5571E-01	7,2827E-02	5,8879E-02
MPV	6,1366E-01	4,5304E-01	2,2710E-01	1,8561E-01	5,7608E-01	7,3303E-01
PDW	6,3642E-02	4,2905E-03	5,2800E-02	1,0399E-01	1,5952E-02	2,2325E-02
PCT	3,8762E-01	4,1714E-01	8,9590E-01	8,8894E-01	3,6580E-01	1,2145E-01
LPCR	4,2821E-01	2,0688E-01	4,0600E-01	4,2290E-01	2,3266E-01	1,3500E-01
WBC	1,0513E-01	2,0955E-02	8,6900E-02	1,0459E-01	1,0754E-01	6,9213E-02
HGB	2,1103E-03	3,4636E-03	4,7000E-02	4,5351E-02	1,1401E-03	1,0254E-03
MCH	8,5162E-02	1,6679E-01	9,8200E-02	7,8596E-02	6,8396E-02	2,0015E-02
MCHC	2,7073E-02	9,6814E-04	5,0020E-01	4,9520E-01	2,2732E-02	1,7891E-03
LYM	5,2710E-01	6,6189E-01	1,8310E-01	2,1358E-01	5,2914E-01	8,6178E-01
GRAN	2,5800E-03	1,9648E-03	1,1250E-01	2,5231E-02	7,6518E-04	1,8860E-04
MID/Mon	9,6808E-01	9,2593E-01	2,9040E-01	2,6839E-01	9,7172E-01	7,3656E-01
LYM%	2,0219E-03	9,2005E-03	4,9790E-01	5,6903E-01	2,1835E-03	9,8892E-03
GRA%	9,4053E-04	6,4821E-03	1,8130E-01	8,1910E-03	9,4064E-05	5,6383E-04
MID%/Mon%	7,7077E-01	5,7091E-01	6,4140E-01	7,0677E-01	6,2504E-01	4,0509E-01
BChE	6,9030E-05	8,6869E-05	1,6600E-02	1,2641E-02	4,6110E-05	9,2393E-05
PON1	1,2470E-01	1,7453E-01	3,3940E-01	3,3442E-01	1,0913E-01	1,9118E-01
ALT	3,9147E-01	7,0025E-01	6,2230E-01	6,4448E-01	4,8824E-01	3,6785E-01
AST	1,1519E-01	1,4500E-01	8,4200E-02	8,9263E-02	1,1324E-01	7,2283E-02
ALB	2,7941E-10	4,8232E-04	1,0000E-04	1,6100E-04	4,0000E-10	1,4822E-05
Glu	1,4298E-06	1,4424E-04	4,0290E-01	3,3060E-01	5,0090E-07	4,8000E-09
GGT	1,2528E-01	3,2373E-01	3,0450E-01	3,0402E-01	1,2868E-01	5,1474E-02
Tot.Prot	1,4225E-02	2,0200E-02	7,6390E-01	8,1158E-01	9,4529E-03	4,8213E-03
PHOS	9,5336E-01	9,5530E-01	6,0610E-01	6,5200E-01	9,5567E-01	9,4775E-01
UREA	5,2244E-01	4,3262E-01	1,5700E-02	1,9434E-02	4,1219E-01	2,3446E-01
TRIGS	5,4092E-01	2,7576E-01	4,6360E-01	4,4415E-01	4,4995E-01	3,1072E-01

CREA	8,2687E-01	6,9288E-01	7,8670E-01	7,4201E-01	8,5630E-01	3,6023E-01
ALP	4,6137E-03	6,7308E-03	4,0000E-04	2,9100E-04	4,1194E-03	2,4783E-03
K-GUV	2,7468E-01	2,3002E-01	6,0100E-02	6,6990E-03	8,0799E-02	2,0911E-03
BilAc	6,5471E-01	3,3276E-01	6,4300E-02	1,9990E-01	6,1561E-01	1,4517E-01
HDL	3,0541E-03	2,3157E-03	1,1870E-01	1,0509E-01	3,1937E-03	4,8701E-03
LDL	9,7080E-05	3,4208E-06	3,5000E-03	3,9680E-03	8,1024E-05	1,4952E-04
CK-NAC	5,1357E-03	6,7553E-03	5,3160E-01	5,5540E-01	5,4838E-03	1,7477E-03
Chol	4,2202E-05	1,2984E-05	9,3600E-02	1,4779E-01	3,2150E-05	7,5430E-05
LDH	4,3388E-04	8,5892E-06	6,6000E-03	5,7530E-03	4,1411E-04	1,1932E-03
Ur. Acid	8,9246E-03	4,2259E-03	1,0590E-01	1,9230E-01	1,0577E-02	8,1855E-03
LAC	2,6645E-01	2,2781E-01	3,8910E-01	3,4846E-01	2,9935E-01	2,6175E-01
NEFA	1,6336E-03	1,1740E-03	5,2590E-01	4,7766E-01	1,8790E-03	2,9905E-03
Transf	1,1429E-10	5,5512E-09	1,7730E-01	1,5974E-01	1,0000E-10	7,1000E-08
Fe	6,9375E-04	6,3257E-10	1,0300E-02	1,0332E-02	7,8995E-04	1,1299E-05
a1-AGP	1,8778E-02	3,9015E-02	3,1360E-01	3,4780E-01	2,3781E-02	1,2017E-02

Supplementary Table S2. Distributional comparison of key biomarkers between the training cohort and the independent cohort for out-of-distribution assessment. Only biomarkers present in both the training cohort and the independent cohort were included; features were selected based on availability in the independent cohort.

**Feature name**	**Mean ± SD (training cohort, n=20)**	**Mean ± SD (external cohort, n=28)**	**Median [Q1-Q3] (training cohort, n=20)**	**Median [Q1-Q3] (external cohort, n=28)**
WBC	7,22 ± 1,47	7,62 ± 3,93	6,95 [5,98 - 8]	7,15 [5,88 - 7,75]
LYM	2,13 ± 0,79	1,77 ± 0,65	2 [1,65 - 2,8]	1,6 [1,3 - 2,15]
MID/Mon	0,52 ± 0,13	0,51 ± 0,15	0,5 [0,4 - 0,6]	0,5 [0,4 - 0,6]
GRAN	4,57 ± 1,48	5,34 ± 3,88	4,4 [3,2 - 5,63]	4,75 [3,55 - 5,5]
HGB	118,57 ± 15,98	114,96 ± 17,02	122 [109 - 128]	116 [106,5 - 124,25]
MCH	30,27 ± 2,35	30,04 ± 1,55	30,35 [28,95 - 31,2]	29,9 [29,18 - 31,05]
MCHC	346,1 ± 8,66	336,54 ± 9,17	346,5 [340,5 - 351,25]	337 [334 - 341]
RBC	3,91 ± 0,48	3,84 ± 0,6	3,96 [3,6 - 4,26]	3,86 [3,39 - 4,17]
MCV	87,21 ± 6,36	89,28 ± 5,08	88,45 [82,55 - 90,33]	88,9 [86,75 - 91,4]
RDWa	66,32 ± 6,8	62,02 ± 5,12	66,6 [61,25 - 70,43]	61,1 [58,8 - 64]
HCT	34,33 ± 4,75	34,24 ± 5,31	34,9 [32,48 - 36,82]	34,35 [31,1 - 37,15]
RDW%	19,18 ± 1,32	17,41 ± 0,87	18,9 [18,08 - 19,73]	17,25 [16,7 - 17,73]
PLT	255 ± 78,76	258,71 ± 96,85	243,5 [203 - 295,25]	246,5 [214,75 - 287,5]
MPV	8,61 ± 0,96	8,21 ± 0,78	8,55 [7,98 - 9,13]	8,25 [7,78 - 8,7]
PDW	12,45 ± 1,31	11,91 ± 1,1	12,35 [11,5 - 13,15]	12,05 [11,2 - 12,45]
LPCR	18,78 ± 5,86	16,86 ± 5,22	18,8 [14,35 - 22,08]	16,8 [13 - 19,78]
PCT	0,21 ± 0,06	0,21 ± 0,07	0,21 [0,18 - 0,25]	0,19 [0,17 - 0,24]
ALT	25,76 ± 20,65	13,35 ± 22,32	21,35 [17,35 - 25,7]	7,35 [4,5 - 10,05]
AST	27,4 ± 12,54	25,23 ± 35,63	25 [21,75 - 27,25]	16,1 [12,85 - 20,68]

GGT	50,5 ± 84,91	26,93 ± 33,01	27 [21 - 41]	12,8 [10 - 28,65]
Glu	4,53 ± 1,28	4,64 ± 0,94	4,3 [3,9 - 4,55]	4,59 [3,98 - 4,96]
ALB	38,72 ± 4,88	33,38 ± 7,2	40,3 [35,75 - 42,4]	33,15 [27,8 - 37,33]
Fe	16,03 ± 4,9	12,24 ± 4,43	15,05 [13,43 - 17,03]	12,85 [8,4 - 14,35]
CREA 2R	84,5 ± 12,17	94,46 ± 35,59	85 [74,75 - 89,25]	89,5 [70 - 98,5]
Ca 2R	2,62 ± 0,24	2,22 ± 0,3	2,7 [2,53 - 2,8]	2,25 [2,04 - 2,39]
CK-NAC	95,85 ± 74,06	62,04 ± 55,45	72,5 [37,5 - 116,75]	36,5 [22 - 79,75]
LDH L-P	259,4 ± 77,68	266,57 ± 97,93	245 [206,25 - 295,25]	243 [202,5 - 313,75]
UREA 1R	4,98 ± 2,38	7,03 ± 4,13	4,41 [3,28 - 6,08]	5,69 [4,08 - 9,39]
TP	76,53 ± 8,99	65,85 ± 10,67	78,6 [74,05 - 80,88]	65,3 [56,98 - 72,95]
TRIGS	1,32 ± 0,91	1,37 ± 0,54	1,08 [0,81 - 1,31]	1,28 [0,93 - 1,69]
PHOS	1,23 ± 0,29	1,26 ± 0,25	1,23 [1,1 - 1,4]	1,21 [1,15 - 1,31]
Chol	4,37 ± 0,92	4,16 ± 1,33	4,42 [3,87 - 5,21]	4,07 [3,25 - 4,89]
ALP	196,98 ± 16,53	315,21 ± 285,91	195,75 [192,13 - 198,25]	229,5 [187,5 - 300]
BChE	1854,07 ± 815,74	5562,96 ± 2093,37	1842,16 [1513,27 - 2207,74]	5087 [4188,75 - 6155,5]
HDL R	1,2 ± 0,24	1,09 ± 0,45	1,19 [1,06 - 1,43]	0,99 [0,8 - 1,28]
LDL R	2,7 ± 0,77	1,98 ± 0,89	2,67 [2,35 - 3,45]	1,94 [1,5 - 2,64]

Supplementary Table S3. Population Stability Index (PSI) for key biomarkers between the training cohort and the independent cohort (4 bins).

Feature name	PSI	Feature name	PSI	Feature name	PSI
WBC	0.2045	PLT	0.1546	Ca 2R	4.6216
LYM	0.5058	MPV	0.1199	CK-NAC	0.1561
MID/Mon	0.0276	PDW	0.1875	LDH L-P	0.0295
GRAN	0.0715	LPCR	0.1270	UREA 1R	0.2472
HGB	0.4493	PCT	0.0797	TP	4.1882
MCH	0.0290	ALT	2.7170	TRIGS	0.2832
MCHC	0.8825	AST	1.2445	PHOS	0.2696
RBC	0.1546	GGT	2.6373	Chol	0.1905
MCV	0.4475	Glu	0.4511	ALP	3.9345
RDWa	1.0455	ALB	0.6789	BChE	5.9256
HCT	0.0583	Fe	0.3257	HDL R	0.4039
RDW%	2.5532	CREA 2R	0.4706	LDL R	0.8380
